# Effects of Nitroxin and arbuscular mycorrhizal fungi on the agro-physiological traits and grain yield of sorghum (*Sorghum bicolor* L.) under drought stress conditions

**DOI:** 10.1371/journal.pone.0243824

**Published:** 2020-12-28

**Authors:** Shirzad Kamali, Ahmad Mehraban

**Affiliations:** Department of Agriculture, Zahedan Branch, Islamic Azad University, Zahedan, Iran; United Arab Emirates University, UNITED ARAB EMIRATES

## Abstract

The use of bio-fertilizers in agro-ecosystems is considered to have the potential to improve plant growth in extreme environments featuring water shortages. However, while arbuscular mycorrhizal fungi (AMF) and bacteria bio-fertilizers have been used in other plants to enhance stress tolerance, little is known about their symbiotic effect on sorghum (*Sorghum bicolor* L.) growth under drought stress conditions. Therefore the aim of this study was to investigate the inoculation of sorghum with Nitroxin and *Glomus mosseae* and their interaction effects on the agro-physiological characteristics and grain yield of sorghum under drought stress conditions. Nitroxin is a bio-fertilizer that consists of a mixture of Azospirillum and Azotobacter bacteria. The results showed that co-inoculation of sorghum seeds with Nitroxin and AMF improved the chlorophyll (a, b and total) content, soluble proteins, water use efficiency) WUE(, relative water content (RWC), nitrogen (N) content in the plant, AMF spore density, proline content, grain yield, panicle length, the number of panicles per plant, grain number per panicle, 1000-grain weight and decreased the electrolyte leakage and water saturation deficit (WSD) in drought stress and non-stress conditions. Under drought stress conditions, there was a 27% increase in grain yield under the synergistic effects of bacteria and fungi compared to the non-application of these microorganisms. The results of this experiment show that Nitroxin and AMF bio-fertilizers can mitigate the negative effects of stress on plants in drought stress conditions by increasing the amount of photosynthetic pigments, soluble proteins and osmotic regulation and decreasing electrolyte leakage. We found that the combination of bacteria and AMF for sorghum growth and yield increment is a promising method to cope with the stress caused by drought.

## Introduction

Drought stress is a challenging issue for crop production problems in arid and semi-arid regions of the world [1]. This type of stress reduces the growth and yield of plants due to the decreasing water content and changes in some of the physiological and metabolic characteristics. Water deficit stress can inhibit photosynthesis, alter the chlorophyll content and damage the photosynthetic apparatus through the stomatal closing, thus preventing carbon dioxide from entering the chloroplast. This is as well as the reduction in cellular water potential [2]. The accumulation of reactive oxygen species (ROS) is another biochemical change that takes place in plants under drought stress conditions. ROS are highly toxic and reactive and can greatly disrupt the normal metabolism of cells in the absence of protective mechanisms in plants [3]. ROS can cause oxidative stress and lipid peroxidation (membrane damage), destruction of proteins and photosynthetic pigment, the disruption of RNA and DNA functions, and result in serious damage to the cells and structures of the plants. As a result, it may reduce the leaf area of the plant which affects the root and shoot growth of the plant in turn [2].

Some of the methods used for enhancing the ability to combat drought stress in plants are the application of stored water, traditional breeding methods and the genetic alteration of plants. The application of these techniques in terms of methodology and implementation is very complex and laborious. The utilization of biological fertilizers is an alternate approach to plant growth and production under drought stress conditions. Bio-fertilizers have been applied in agriculture to significantly reduce the use of chemical inputs under stress conditions and it is used as a strategy for reducing the effects of stress on most crops under drought stress conditions. The symbiotic relationship between plants and symbiotic species can reduce the negative effects of drought stress on the plant by strengthening the root system [4]. The arbuscular mycorrhizal fungus (AMF) is a type of bio-fertilizer that receives sugars, amino acids, vitamins, and other organic substances from the host plant. In turn, it absorbs minerals, especially phosphate from the soil which can improve the growth and yield of the host plant [5]. AMF improves plant production under drought stress conditions through the absorption of non-mobile nutrients such as phosphorus, zinc and copper. It increases the tolerance of the plants to drought stress by improving the water absorption and leaf water potential, controlling the stomatal pores and transpiration and extending the length and depth of the roots leading to the development of terminal hyphae [6]. Therefore the hydraulic conductivity of the root system of mycorrhizal plants is more than that in non-mycorrhizal plants because of the wider root surface area and root length of the plants. As a result, the water conductivity per unit length of the roots in mycorrhizal plants increases by 2–3 times compared with non-mycorrhizal plants. The leaves of mycorrhizal plants have less resistance to water vapor diffusion than non-mycorrhizal plants and the leaf area of mycorrhizal plants is also expanded compared with non-mycorrhizal plants [7].

In this regard, Nxele et al. [8] reported an increase in hydrogen peroxide content and a decrease in the amounts of chlorophyll, height and the dry weight of sorghum under drought stress conditions. An increase in the content of chlorophyll (a, b, and total) in maize under inoculation with AMF and drought stress conditions was reported by Xu et al. [9]. In a study to investigate the effects of Nitroxin inoculation on millet traits, Darbani et al. [10] reported higher amounts of soluble carbohydrates and the relative water content in the leaves of this plant under inoculation and stress conditions. Farnia and Hadadi [11] showed that by increasing the irrigation interval from 7 to 21 days, the values of 1000-grain weight, chlorophyll index, grain yield, and harvest index of maize decreased while the inoculation of this plant with AMF increased the amounts of these traits under stress conditions. An increase in plant growth, biomass production, the activity of antioxidants, soluble sugar and the amelioration of salt stress by AMF was reported by Wang et al. [12] for sweet sorghum. Shi et al. [13] also showed that inoculation of sorghum plants with AMF improved the nutrient uptakes of P, N and S, and the photosynthesis efficiency of the plant in Mo-contaminated soil.

Nitroxin is another bio-fertilizer that contains Azospirillum and Azotobacter bacteria [14]. These bacteria can increase nitrogen fixation, phosphate solubilization, iron release from the soil and siderophore production as well as the presence of plant hormones such as auxin, cytokinin, gibberellin, and ACC-deaminase [15]. In one study, Lamochi and Sakinejad [16] found that the inoculation of maize with Nitroxin increased both the grain and biological yield of the plant. Bacteria known as mycorrhiza helper bacteria (MHB) can have a positive effect on the establishment and operation of mycorrhizal symbioses [17]. MHB may improve the formation and establishment of mycorrhiza, specifically the association between plant roots and AMF, through various mechanisms such as the production of AMF-stimulating substances/nutrients and/or the modification of root exudates and/or stimulating the plant host to produce substances that improve the formation of mycorrhiza. Plants, AMF and bacteria can therefore be considered tripartite associations leading to a consortium promoting plant growth [18]. The interaction between the micro-organisms in the rhizosphere influences plant growth and production and health directly or indirectly in agricultural and natural habitats by providing nutritional elements and resistance to biotic and abiotic stresses [19]. Therefore these symbiotic relationships between the soil microbes are known to have an advantageous effect on plants by improving the accessibility of the nutrients to the host plants and inducing the plant defenses against different stresses such as drought and salinity [17].

Some studies have shown synergistic microbial interactions between rhizobacteria and AMF in the growth of plants. In an extensive study, Javan Gholiloo et al. [20] reported that the synergistic relationships between mycorrhiza, Azotobacter and Azospirillum have a positive effect on the growth and tolerance of valerian to drought stress. Their results showed that the interaction effect of both factors on the root and shoot dry weight, proline, carotenoids, nitrogen and potassium and essential oils were significant. The main effects of these factors on RWC, chlorophyll a and b, phosphorus and soluble sugars content were significant. In another study, Harishkumar et al. [21] showed that the application of Azospirillum, Azotobacter, phosphate solubilizing bacteria (PSB) and AMF increased the number of branches, number of leaves, plant height, phosphorus content and nitrogen content of basil compared to the control treatment. The results of their experiment revealed incremental effects because of the dual matchable mixtures of inoculants due to their powerful synergistic association with each other. In the study by Patil and Santhosh [22], the number of productive tillers/hill, panicle length, number of filled and unfilled grains/panicle, grain yield, spore count and root colonization of rice plants were significantly increased following the combined inoculation of AMF+ Azospirillum compared to the control and individual inoculations. Rahimi et al. [23] reported that the highest grain yield, biological yield, oil percentage yield, 1000-grain weight, chlorophyll (a, b and total) content, carotenoid content, total phenols, total flavonoids and DPPH antioxidant activity of cephalaria (*Cephalaria syriaca* L.) were observed following a dual inoculation with AMF + Azotobacter treatment.

Sorghum is a plant in the cereal family that is cultivated for human consumption, animal feed and fuel [24]. Specifically, it is the world's fifth most important cereal after wheat, rice, corn and barley [25] but little research has been carried out on enhancing the role of bio-fertilizers in relation to the growth and yield of sorghum, particularly under drought stress conditions. Regarding the benefits of the microbial collaboration, unfortunately there have been no previous studies on the simultaneous inoculation of sorghum with AMF and Azospirillum and Azotobacter examining their interaction effects on plant growth and their potential to improve the stress tolerance of the plant under drought stress conditions. In the southern region of Saravan, Iran, drought stress threatens the growth of the crops due to low rainfall. The sorghum that grows in this region is an important plant and its yield is significantly affected each year by drought stress. Therefore in this study, we examined the impact of AMF, Nitroxin and their combination on the plant yield and physiological indicators.

## Materials and methods

### Experimental site and conditions

This experiment was conducted on a private land in the region of Saravan, Sistan and Baluchestan province, Iran during the sorghum growing season (June–September) in 2017 and 2018. The Saravan region is located at 27°37′ N latitude and 62°20′ E longitude with an elevation of 1165 m above the mean sea level. The area has a dry climate with an average annual precipitation of 100 mm. The monthly precipitation as well as the temperature of the two study seasons (2017 and 2018) has been shown in Table 1.

**Table 1:** Meteorological parameters for the field site during the sorghum growing season in 2017 and 2018.

Month	Mean air temperature (Ċ)		Mean rainfall (mm)	
	2017	2018	2017	2018
**June**	32.6	31.9	3.2	0
**July**	32.5	33	1.6	1.2
**August**	31.6	33	0	0
**September**	29	27.8	0	0

Each experimental plot size was 5 m × 3 m and there were 5 planting rows in each plot. The distance between the blocks was also 3 m and the distance between the plots was 1 m. According to the physical and chemical properties of the soil in the research field (Table 2), all phosphorous (225 kg ha^−1^ in the form of superphosphate) and potassium (225 kg ha^−1^ in the form of potassium sulfate) fertilizers were applied as the basal dose at the time of seedbed preparation.

**Table 2:** Physical and chemical properties of the soil in the research field.

Soil texture	Potassium	Nirogen available	Phosphorous	OC	PH	EC
	(ppm)	(%)	(ppm)	(%)		(ds/m)
Sandy	40	0.01	2.2	0.02	8.52	0.83

Nitrogen fertilizer (350 kg ha^−1^ in the form of urea) was applied as ½ at sowing and ½ at the stage of 6–8 leaves. The sorghum cultivar ‘Payam’ seeds were planted on June 1st in 2017 and 2018 with planting densities of 20 plants m^2^ at depths of 2–5 cm and 0.10 m distance in the row. After the establishment of the plants, the irrigation treatments were applied based on evaporation from an evaporation pan. The method used for pest and disease control was based on the common recommendations in this region. Harvest was conducted on 27th September 2017 and 29th September 2018.

### Experimental design and treatments

This experiment was conducted as a factorial experiment using a randomized complete block design with four replications. The first factor consisted of three irrigation levels including irrigation after 60 mm evaporation (non-stress), 120 mm (moderate drought stress) and 180 mm evaporation (severe drought stress) from a class A evaporation pan. The second factor consisted of four levels of bio-fertilizer application (1- non-application or control, 2- inoculation with Nitroxin, 3- inoculation with AMF and 4- co-inoculation with Nitroxin and AMF).

The Nitroxin bio-fertilizer that was used in this study contained 1 × 10^7^ living cells forming a set of the most effective nitrogen-fixing bacteria including the genus Azotobacter and Azospirillum produced by the Asian Institute of Biotechnology, in Iran. The sorghum seeds were inoculated with Nitroxin bio-fertilizer in the absence of light before planting. AMF (*Glomus mosseae*) was purchased from the Zist Fanavar Turan corporation in Iran which contained 200 active spores per gram. Accordingly, before sowing the sorghum seeds in the plots following the fungal treatment, 5g of inoculum was poured into the holes that were prepared for the sowing of the seeds. Soil was then added to the inoculum with the seeds put on it. Finally, the seeds were covered with soil.

### Sampling and measurement

In the mid-flowering stage, fresh leaves were randomly collected and immediately frozen in liquid nitrogen. They were quickly taken to the laboratory and stored at −80°C until the physiological analysis.

#### Measurement of the leaf water status (RWC and WSD)

The relative water content (RWC) and water saturation deficit (WSD) of the sorghum leaves were measured using the Omae et al.’s [26] method. For each treatment, a fully developed fresh leaf of sorghum was randomly selected. All samples from the treatments were put into plastic bags which were transferred into a box of ice and then immediately transferred to the laboratory. Eight discs were made from the middle of the leaf using a sharp cork borer. The exact weights of the fresh discs were determined and recorded for each experimental plot. After weighing, the discs were transferred to the Petri dishes containing distilled water and kept in the dark for 18 hours. Afterward, the discs were dried using a paper drier and their turgid weight was measured. The weighted samples were then placed in paper bags and transferred to an oven at 80°C for 24 h. Finally, the dry weights of the samples were obtained used accurate scales and then the RWC and WSD of the sorghum leaves were calculated using the following formulas.

$RWC\;(\%)=\frac{\left(FW-DW\right)}{\left(TW-DW\right)}\times 100$

WSD(%)=100−RWC

RWC: relative water content, FW: fresh weight, Tw: turgid weight, and: DW: the dry weight of discs.

#### Water use efficiency (WUE) measurement

Water use efficiency is described as the yield achieved per unit of water applied to the plant under evaluation. The WUE was then determined on the basis of the total amount of water applied. The total grain yield was obtained as follows [27]:

$$WUE\;(mg/kg)=\frac{Total\;grain\;yield}{Total\;water\;applied}\times 100$$

#### Chlorophyll (a, b and total) content measurement

To measure the chlorophyll content of the sorghum leaves according to Lichtenthaler's method [28], 100 mg of fresh tissue of leaf was ground with 15 mL of 80% acetone. The extract was then filtered using filter paper and the solution was transferred into a volumetric flask. The final solution volume reached 10 mL using 80% acetone. After 24 hours, the solution was transferred to centrifuge tubes and was centrifuged at a speed of 6,000 rpm for 10 min. The absorbance of the final solution was then measured by spectrophotometer at wavelengths of 647, 663 and 470 nm. The concentrations of chlorophyll a, chlorophyll b and total chlorophyll were then calculated based on the following formulas. In these formulas, A_Abs 663_, A_Abs647_, and A_Abs470_ are the absorbances of the final solution at wavelengths of 663, 647 and 470 nm respectively.

Chlorophylla=12.25AAbs663–2.79AAbs647

Chlorophyllb=21.5AAbs647–5.1AAbs663

ChlorophyllTotal=Chlorophylla+Chlorophyllb

#### Measurement of nitrogen (N) content in plant

The Kjeldahl method was used to determine the nitrogen concentration of the sorghum leaf tissue [29]. In the presence of a catalyst, 1 g of ground leaf tissue was digested in 8 mL of concentrated sulfuric acid. Then, using a semiautomatic device, sodium hydroxide was applied to distill the sample. In order to extract the nitrogen, 4% boric solution was used. In the presence of an indicator (bromocresol green and methyl red), titration was performed with sulfuric acid. The following formula was used to calculate the concentration of total nitrogen:

$N\;(\%)=(\frac{V\;(H2SO4)\times N\;(H2SO4)}{EC2})\times 100$

V(H_2_SO_4_): volume of H_2_SO_4_ used for titration

N(H_2_SO_4_): normality of H_2_SO_4_ used for titration

SW: the dry weight of leaf tissue.

#### Soluble protein content measurement

The concentration of soluble protein in the sorghum leaves was measured using Bradford's method [30]. In this method, 1g of sorghum leaf tissue was ground using 4 mL of sodium phosphate buffer (0.1 mM ethylenediaminetetraacetic acid (EDTA), 100 μM phenylmethylsulfonyl fluoride (PMSF), and 2% polyvinylpyrrolidone (w/v)) with pH 7.2. The resulting mixture was centrifuged at 15,000 rpm for 30 min. After that, 0.1 mL of supernatant was taken and 5 ml of Bradford reagent was added. The solution was mixed well and kept aside for 15 min and then the absorbance of the final solution was measured using a spectrophotometer at wavelengths of 595 nm. The protein concentration was determined using bovine serum albumin (BSA) as a primary standard and protein concentration before being expressed as mg g^-1^ FW of leaf fresh tissue.

Bradford reagent preparation: 100 mg of Coomassie brilliant blue (G 250) was dissolved in 50 mL of 95% ethanol before 100 mL of orthophosphoric acid was added and made up to 200 mL using distilled water. Following this, 1 ml of dye solution was taken and 4 ml of distilled water was added before being used for the sample analysis.

#### Proline content measurement

The content of proline in the leaf samples was determined using Bate’s method [31]. Fresh leaf samples (0.5 g) were homogenized in 10 mL of 3% sulphosalicylic acid before being centrifuged at 1200 g for 10 min. A 2-mL supernatant was mixed with 2 mL acid ninhydrin reagent and 2 mL glacial acetic acid. Afterward, the samples were incubated at 100°C for 60 min. Before adding 4 mL toluene to each sample, the sample materials were cooled in an ice bath. The toluene layer was read at 520 nm with a spectrophotometer.

#### Electrolyte leakage measurement

The leakage of electrolytes into the leaves was measured using an electrical conductivity meter as described by Saadalla et al. [32]. Leaf discs (1 cm in diameter) from two randomly chosen plants per replicate were taken from the middle portion of the youngest fully developed leaf and placed in individual stoppered vial containing 20 mL of distilled water after three washes with distilled water to remove any surface contamination.

The samples were incubated at room temperature (25°C) on a shaker (100 rpm) for 24 hours. The electrical conductivity of samples (EC1) was measured after incubation. The same samples were then placed in an autoclave at 120°C for 20 min and the second measurement (EC2) was done after cooling the solution to room temperature. Eventually, electrolyte leakage was calculated using the following formula and expressed as a percentage.

$Electrolyte\;leakage=(\frac{EC1}{EC2})\times 100$

EC1: conductivity reading at room temperature

EC2: conductivity reading at 120°C.

#### AMF spore density measurement

To calculate the AMF spore density in the soil, according to the method used by Daniels and Skipper [33], 20 g of dried soil was wet sieved and suspended in 20 mL of water in a 50-mL conic tube. A 25-mL sucrose solution (70% v/w) was injected into the bottom of the tube, forming a stepped density gradient that was centrifuged at 900 g for 3 min. The AMF spores were collected from the interface between the sucrose solution and water, washed with tap water on a 36 μm sieve for 2 min and transferred to a 50-mL conic tube with 10 mL of water. Finally, the conic tube was vortexed and 10 successive aliquots of 100 μL were immediately transferred to Petri dishes to count and identify the spores under a stereomicroscope. The number of viable spores was finally expressed per 100 g of soil.

#### Yield and yield components

Once the sorghum plants reached physiological maturity, the plants that were 1 m^2^ from each plot were harvested for the measurement of their panicle length, the number of panicles per plant and the grain number per panicle. The grain yield and 1000-grain weight of the plant were calculated using precise scales after drying the grains to 14%.

### Statistical analysis

Microsoft Excel was used to process the data. Variance analysis (ANOVA) was performed using the SAS (version 9.2) statistical software package (SAS, Cary, NC) to test the effects of the bio-fertilizers and irrigation treatments. The least significant difference (LSD) test at the 0.05 probability level was used to compare the means of the measured traits.

## Results

### Chlorophyll (a, b, and total) content

The results of the combined analysis of variance (Table 3) showed that the effects of irrigation and the application of the bio-fertilizers, as well as the interaction effects of these treatments on the content of chlorophyll (a, b, and total), were significant (p≤0.01). The results of the means comparison showed that increasing drought stress levels significantly decreased the chlorophyll content of the sorghum plants while all treatments of inoculation with the bio-fertilizers improved the chlorophyll content. The highest amounts of chlorophyll a, chlorophyll b and the total chlorophyll of grain sorghum (0.704 ± 0.002, 0.197 ± 0.0005 and 0.90 ± 0.002 mg g^-1^ FW respectively) were obtained from the co-inoculation with AMF and Nitroxin under irrigation after 60 mm evaporation (non-stress) conditions. The lowest amounts of chlorophyll a and total chlorophyll (0.23 ± 0.001 and 0.29 ± 0.001 mg g^-1^ FW respectively (were obtained from the interaction effects of non-inoculation with AMF and Nitroxin and irrigation after 180 mm evaporation (severe drought stress) treatments. Moreover, the lowest amount of chlorophyll b content (0.065 ± 0.0004 mg g^-1^ FW (was obtained from the interaction effect of non-inoculation with these bio-fertilizers and irrigation after 180 mm evaporation (severe drought stress) which showed no significant difference compared to the interaction effect of AMF application and irrigation after 180 mm evaporation (0.069 ±0.0009 mg g^-1^ FW ((Table 4).

**Table 3:** Combined analysis of variance for some traits of grain sorghum during two years.

Sources of variates (S.O.V)	Degrees of freedom (df)	Mean squares (MS)					
		Chlorophyll a	Chlorophyll b	Total chlorophyll	Soluble protein	RWC	WSD
**Year (Y)**	1	0.0005894^ns^	0.00004116^ns^	0.0009421^ns^	0.0015553^ns^	14.473^ns^	14.47^ns^
**Replication (Year)**	6	0.0009705	0.00016470	0.0018368	0.0008522	44.604	44.60
**Irrigation (I)**	2	0.9362751**	0.05247866**	1.4313352**	0.5536226**	5586.744**	5586.74**
**Bio-fertilizers) B(**	2	0.0834731**	0.00793511**	0.427974**	0.0378909**	380.042**	380.04**
**I×B**	4	0.0066925**	0.00112138**	0.0087003**	0.0064401**	55.659**	55.65**
**Y×I**	2	0.0000231^ns^	0.00000130^ns^	0.0000354^ns^	0.0000196^ns^	0.256^ns^	0.256^ns^
**Y×B**	2	0.0000020^ns^	0.00000020^ns^	0.0000035^ns^	0.0000013^ns^	0.012^ns^	0.012^ns^
**Y×I×B**	4	0.0000001^ns^	0.00000003^ns^	0.0000002^ns^	0.0000002^ns^	0.009^ns^	0.009^ns^
**Error**	48	0.0003253	0.00010999	0.0007198	0.0008943	17.266	17.26

Ns, * and ** represent non-significant and significant at the 5% and 1% levels of probability, respectively.

**Table 4:** Means comparison of the interaction effects of irrigation and bio-fertilizers on some traits of grain sorghum.

Traits Treatments		Chlorophyll a	Chlorophyll b	Total chlorophyll	Soluble proteins	RWC	WSD
		(mg/g FW)	(mg/g FW)	(mg/g FW)	(mg/g FW)	(%)	(%)
**After 60 mm evaporation (non-drought stress)**	**Control**	0.626±0.018c	0.140±0.0017c	0.76±0.008c	0.79±0.007a	85.49±1.8b	14.50±0.9f
	**AMF**	0.665±0.001b	0.170±0.0006b	0.83±0.002b	0.80±0.001a	87.36±0.2ab	12.63±0.5fg
	**Nitroxin**	0.667±0.007b	0.170±0.0004b	0.83±0.007b	0.79±0.001a	88.86±0.7ab	11.13±0.6fg
	**AMF+Nitroxin**	0.704±0.002a	0.197±0.0005a	0.90±0.002a	0.81±0.002a	90.24±0.2a	9.76±0.4g
**After 120 mm evaporation (moderate drought stress)**	**Control**	0.437±0.005g	0.126±0.0022de	0.56±0.007g	0.62±0.002de	72.45±1.3d	27.54±0.5d
	**AMF**	0.467±0.001f	0.133±0.0004cd	0.60±0.001f	0.64±0.001d	74.14±0.1d	25.85±0.3d
	**Nitroxin**	0.538±0.004e	0.138±0.0007c	0.67±0.004e	0.71±0.003c	78.43±0.2c	21.56±0.7e
	**AMF+Nitroxin**	0.572±0.003d	0.143±0.0011c	0.71±0.005d	0.74±0.003b	79.63±0.3c	20.36±0.3e
**After 180 mm evaporation (severe drought stress)**	**Control**	0.230±0.001j	0.065±0.0004g	0.29±0.001j	0.47±0.001h	52.19±0.1g	47.81±0.6a
	**AMF**	0.284±0.003i	0.069±0.0009g	0.35±0.004i	0.52±0.002g	62.21±0.2f	37.78±0.3b
	**Nitroxin**	0.354±0.002h	0.101±0.0008f	0.45±0.004h	0.56±0.002f	63.89±0.2f	36.10±0.4b
	**AMF+Nitroxin**	0.426±0.001g	0.119±0.0004e	0.54±0.003g	0.60±0.001e	68.13±1.6e	31.86±0.3c

Within a column, means ± SE with similar letters are not significantly different at 5% probability based on LSD test.

### Soluble protein

As the analysis of variance (Table 3) shows, the simple effects of irrigation and inoculation with the dual bio-fertilizer treatment (Nitroxin and AMF) and the interaction effects of these treatments on the soluble protein content of grain sorghum were significant (p≤0.01). In this experiment, the application of bio-fertilizers, especially in drought stress conditions, led to an increase in the amount of soluble proteins in the plant. In this respect, the lowest amount of this trait (0.47 ± 0.001 mg g^-1^ FW) was obtained from the interaction effect of the non-application of bio-fertilizers and irrigation after 180 mm evaporation (severe drought stress) treatments and the highest amount of this trait (0.81 ± 0.002 mg g^-1^ FW) was obtained from the interaction effect of the co-inoculation with AMF and Nitroxin and irrigation after 60 mm evaporation conditions (non-stress). There was no significant difference between the interaction effect of the Nitroxin application treatment and irrigation after 60 mm evaporation (0.79 ± 0.001 mg g^-1^ FW (and with the interaction effect of AMF inoculation and irrigation after 60 mm evaporation (0.79 ± 0.001 mg g^-1^ FW (and with the interaction effect of AMF inoculation and irrigation after 60 mm evaporation (0.80 ± 0.001 mg g^-1^ FW) (Table 4). The protein content of sorghum plants decreased by 17% under moderate stress and by 40.50% under severe stress compared to non-stress conditions. Furthermore, the application of both fungus and bacteria under severe stress increased the amount of this trait by 26.65% compared to the non-application of the bio-fertilizers under severe stress.

### Leaf water status (RWC and WSD)

The results of the variance analysis showed that the simple effects of the irrigation and dual bio-fertilizer (Nitroxin and AMF) treatments as well as the interaction effect of the irrigation and bio-fertilizers treatment were significant (p≤0.01) in relation to the relative water content and water saturation deficit of the plant (Table 3).

In our experiment with increasing evaporation levels, the RWC of the sorghum leaves was reduced and the WSD was increased. The application of Nitroxin and AMF enhanced RWC and decreased WSD, especially under severe stress. As a result, the highest relative water content of sorghum leaves (90.24 ± 0.2%) and the lowest amount of WSD (9.76 ± 3.1%) was obtained from the interaction effects of the co-inoculation with AMF and Nitroxin treatment and irrigation after evaporation of 60 mm (non-stress conditions). Nevertheless, the lowest amount of RWC (52.19 ±0.1%) and the highest amount of WSD (47.81 ± 3.6%) was obtained from the interaction effect of the non-inoculation treatment and irrigation after evaporation of 180 mm (Table 4).

### Proline content

The simple effects of the irrigation and bio-fertilizer treatments as well as the interaction effect on the proline content of sorghum showed a significant difference at the 1% level (Table 5). Both treatments of the dual bio-fertilizer application and drought stress led to an increase in the proline content compared to the non-application of bio-fertilizers and non-stress treatments. In this regard, the highest proline content (0.64 ± 0.008 μmol g^-1^ FW) was obtained from the interaction effect of the co-inoculation with AMF and Nitroxin bio-fertilizers and irrigation after 180 mm evaporation (severe stress). The lowest content of this trait (0.17 ± 0.023 μmol g^-1^ FW) was obtained by the non-application of these bio-fertilizers under irrigation after 60 mm evaporation conditions, which showed no significant difference compared to the treatment with AMF application alone (0.18 ±0.003 μmol g^-1^ FW), inoculation with Nitroxin (0.18 ± 0.002 μmol g^-1^ FW) and the simultaneous application of AMF and Nitroxin bio-fertilizers (0.20 ± 0.010 μmol g^-1^ FW) under irrigation after 60 mm evaporation conditions (non-stress) (Table 6). Therefore the application of both Nitroxin and AMF under severe stress increased the amount of this trait by 25.42% compared to non-treated plants under severe stress.

**Table 5:** Combined analysis of variance for some traits of grain sorghum during two years.

Sources of variates (S.O.V)	Degrees of freedom (df)	Mean squares (MS)				
		Proline	WUE	Nitrogen content in plant	Electrolyte leakage	AMF spore density
**Year (Y)**	1	0.004429^ns^	0.00115678^ns^	0.66^ns^	461.9^ns^	20.51^ns^
**Replication (Year)**	6	0.006443	0.00059358	8.53	577.3	203.99
**Irrigation (I)**	2	0.240222**	0.23036904**	4453.00**	59609.0**	36513.66**
**Bio-fertilizers) B(**	2	0.048989**	0.01536420**	458.44**	5080.9**	370601.62**
**I×B**	4	0.010946**	0.00180603**	21.38**	731.0**	12774.24**
**Y×I**	2	0.000366^ns^	0.00001113^ns^	0.16^ns^	18.7^ns^	0.68^ns^
**Y×B**	2	0.000014^ns^	0.00000074^ns^	0.56^ns^	1.4^ns^	6.95^ns^
**Y×I×B**	4	0.000003^ns^	0.00000009^ns^	0.60^ns^	0.3^ns^	0.24^ns^
**Error**	48	0.001738	0.00037231	5.56	155.9	172.40

he 5% and 1% levels of probability, respectively.

**Table 6:** Means comparison of the interaction effects of irrigation and bio-fertilizers on some traits of grain sorghum.

Traits Treatments		Proline	WUE	Nitrogen content in plant	Electrolyte leakage	AMF spore density
		(μmol/g FW)	(Kg/m^3^)	(mg/kg)	(μS/cm)	(spores/ 100 g soil)
**After 60 mm evaporation (non-drought stress)**	**Control**	0.17±0.003h	0.40±0.002i	76.8±1.7c	93.37± 0.93gh	0±3.1g
	**AMF**	0.18±0.003h	0.41±0.002i	77.1±2.3c	87.00±3.90gh	272.99±4.1b
	**Nitroxin**	0.18±0.002h	0.41±0.001i	81.5±1.8b	85.87±3.04gh	0±3.9g
	**AMF+Nitroxin**	0.20±0.010h	0.43±0.001h	83.9±2.1a	83.14±1.26h	289.01±2.4a
**After 120 mm evaporation (moderate drought stress)**	**Control**	0.32±0.007g	0.46±0.003g	64.0±1.7e	147.91±1.14de	0±2.2g
	**AMF**	0.38±0.007f	0.49±0.002f	68.1±1.2d	136.45±4.30e	191.96±3.2d
	**Nitroxin**	0.45±0.008e	0.51±0.002ef	75.0±1.4c	116.71±2.23f	0±4.3g
	**AMF+Nitroxin**	0.49±0.003de	0.52±0.002de	75.6±1.6c	97.74±4.79g	234.99±3.6c
**After 180 mm evaporation (severe drought stress)**	**Control**	0.51±0.003d	0.54±0.001cd	51.0±1.5h	194.29±2.60a	0±2.7g
	**AMF**	0.60±0.005b	0.56±0.001c	55.7±1.6g	180.53±1.77b	116.89±2.2f
	**Nitroxin**	0.55±0.005c	0.60±0.003b	57.5±2.1g	164.49±3.74c	0±3.6g
	**AMF+Nitroxin**	0.64±0.008a	0.62±0.002a	61.5±1.3f	154.34±3.91cd	174.87±2.9e

Within a column, means ± SE with similar letters are not significantly different at 5% probability based on LSD test.

### Water use efficiency (WUE) measurement

In our study, the significant effect of irrigation and inoculation with bio-fertilizers and their interaction on the WUE of sorghum was found as shown in Table 5. The reduction in water requirements following the treatment of irrigation after 60 mm evaporation to irrigation after 180 mm evaporation had a substantial effect on the efficiency of the sorghum plant’s water usage, where irrigation after 180 mm evaporation had the highest efficiency of water usage. This irrigation treatment shows that there is a significant difference compared to the other water requirement treatments. Inoculation with the bio-fertilizers increased the WUE of the sorghum plants examined under both stress and non-stress conditions. The highest value of this trait (0.62 ± 0.02 Kg m^-3^) was obtained from the simultaneous application of AMF and bacteria (Azotobacter and Azospirillum) in conditions of severe drought stress, while the lowest amount of this trait (0.40 ± 0.02 Kg m^-3^) was obtained from the non-application of bio-fertilizers in non-stress conditions. This treatment had no significant difference with the mycorrhiza and Nitroxin application alone under non-stress conditions (0.41 ± 0.02 Kg m^-3^). Under severe drought stress conditions, WUE under inoculation with both AMF and Nitroxin was significantly elevated by 14% compared with that under non-inoculated treatment (Table 6).

### Nitrogen content in plant

According to the results of the ANOVA shown in Table 5, the simple effects of irrigation and the application of the bio-fertilizer treatments and the interaction effect of these treatments on the nitrogen content in sorghum plants showed a significant difference at the 1% level. In our study, the results of the comparison of the means (Table 6) showed that the drought treatment remarkably decreased the nitrogen concentration in the leaf tissue. However, under drought stress, the use of bio-fertilizers enhanced the nitrogen concentration (49%) in the co-inoculated plant with AMF and Nitroxin compared to the non-inoculated plants. The highest nitrogen level (83.9 ± 3.1 mg kg^-1^) in the sorghum leaves was observed in the application of Nitroxin + AMF under irrigation after 60 mm evaporation conditions. While under severe drought stress, the non-application of bio-fertilizers obtained the lowest value for this attribute (51.0 ± 3.5 mg kg^-1^).

### Electrolyte leakage

According to the results in Table 5, the simple effects of irrigation and the dual bio-fertilizer application and the interaction effect of these treatments on the electrolyte leakage of sorghum were significant (p≤0.01). In this study, electrolyte leakage from the cell membrane of the sorghum plant increased when there was a higher level of drought stress from moderate to severe. All treatments of inoculation with bio-fertilizers significantly reduced the amount of this trait. Notably, the highest amount of this trait (194.29 ± 2.60 μS cm^-1^) was obtained by the interaction effect of the non-application of bio-fertilizers and irrigation after 180 mm evaporation treatment (severe drought stress). The lowest electrolyte leakage (83.14 ± 1.26 μS cm^-1^) belonged to the treatment of co-inoculation with AMF and Nitroxin application under irrigation after 60 mm evaporation (non-stress) conditions. In our study, electrolyte leakage increased by 58% under moderate stress and 108% under severe stress. Co-inoculation with both Nitroxin and AMF under severe drought stress conditions decreased the electrolyte leakage from the sorghum tissues by 20.56% compared to the non-application of these bio-fertilizers under severe stress conditions (Table 6).

### AMF spore density

The results in Table 5 show that the AMF spore density of sorghum was affected by the simple effects of the irrigation and bio-fertilizer treatments and also the interaction effect of these treatments at the P≤0.01 level. The results of the comparison of the means (Table 6) showed that under drought and non-drought stress conditions, spores were not observed in the control and the plants inoculated only with Nitroxin. Under the condition of co-inoculation with bio-fertilizers, the negative effects of drought stress levels on this trait were reduced. Accordingly, the highest amount of this trait (289.01 ± 44.4 per 100 g soil) belonged to the treatment of co-inoculation of the sorghum seeds with AMF and Nitroxin under irrigation after 60 mm evaporation (non-stress). In this connection, the spore number in co-inoculated plants with Nitroxin and AMF was 20.20% higher than that of the plants inoculated with AMF alone.

### Number of panicles per plant

The number of panicles per plant of sorghum was affected by irrigation and the bio-fertilizers at the 1% level and it was also affected by the interaction effect of irrigation and bio-fertilizers at the 5% level (Table 7). The results of our experiment show that the number of panicles per plant was significantly influenced by the application of bio-fertilizers and drought stress. Importantly, the highest number of panicles per plant (1.45 ± 0.008) was obtained from the simultaneous application of AMF and Nitroxin bio-fertilizers under irrigation after 60 mm evaporation conditions (non-stress), and the lowest number of panicles per plant (0.90 ± 0.004) belonged to the interaction effect of non-inoculation with these bio-fertilizers and irrigation after 180 mm of evaporation (severe stress). There is no significant difference between the treatment of interaction effect of inoculation with AMF and irrigation after 180 mm of evaporation (0.96 ± 0.006) (Table 8).

**Table 7:** Combined analysis of variance for some traits of grain sorghum during two years.

Sources of variates (S.O.V)	Degrees of freedom (df)	Mean squares (MS)				
		Number of panicles per plant	Grain number per panicle	Panicle length	1000-grain weight	Grain yield
**Year (Y)**	1	0.01773^ns^	5744.92^ns^	2.576^ns^	5.86^ns^	39615.6^ns^
**Replication (Year)**	6	0.00904	6737.35	1.153	8.54	45989.7
**Irrigation (I)**	2	0.11206**	704156.5**	195.531**	1546.59**	7134038.6**
**Bio-fertilizers) B(**	2	0.09465**	42508.39**	15.638**	37.18**	392780.4**
**I×B**	4	0.01141*	9900.18*	1.975*	7.36**	54936.1*
**Y×I**	2	0.00041^ns^	66.77^ns^	0.008^ns^	1.66^ns^	231.9^ns^
**Y×B**	2	0.00006^ns^	2.22^ns^	0.077^ns^	0.40^ns^	160.4^ns^
**Y×I×B**	4	0.00011^ns^	2.17^ns^	0.088^ns^	0.80^ns^	25.8^ns^
**Error**	48	0.00498	4026.88	0.880	2.36	24509.4

Ns, *, and ** represent non-significant and significant at the 5% and 1% levels of probability, respectively.

**Table 8:** Means comparison of the interaction effects of irrigation and bio-fertilizers on some traits of grain sorghum.

Traits Treatments		Number of panicles per plant	Grain number per panicle	Panicle length	1000-grain weight	Grain yield
				(cm)	(g)	(kg/ha)
**After 60 mm evaporation (non-drought stress)**	**Control**	1.34±0.037bc	950.35±6.8abc	19.45±0.47ab	38.49±0.40a	2563.5±42.49ab
	**AMF**	1.39±0.024ab	954.47±7.0abc	19.66±0.36ab	38.68±0.11a	2574.6±44.57ab
	**Nitroxin**	1.37±0.015b	955.95±4.0ab	19.68±0.48ab	38.94±0.38a	2576.9±36.09ab
	**AMF+Nitroxin**	1.45±0.008a	963.06±4.6a	20.22±0.12a	39.11±0.10a	2671.5±12.98a
**After 120 mm evaporation (moderate drought stress)**	**Control**	1.14±0.012f	755.09±7.1de	16.54±0.15de	31.22±0.33c	2010.3±19.40e
	**AMF**	1.23±0.006de	805.94±8.4d	16.99±0.17cd	32.07±0.08c	2214.5±22.98d
	**Nitroxin**	1.19±0.016ef	891.41±3.9c	17.7±0.07c	34.21±0.11b	2352.3±14.52cd
	**AMF+Nitroxin**	1.27±0.009cd	899.11±5.0c	18.96±0.10b	34.31±0.34b	2431.9±13.79bc
**After 180 mm evaporation (severe drought stress)**	**Control**	0.90±0.004h	593.9±2.3h	13.32±0.27h	22.09±0.07e	1441.4±6.06h
	**AMF**	0.96±0.006h	652.10±3.6gh	14.78±0.07g	25.10±0.13d	1670.3±10.82g
	**Nitroxin**	1.06±0.007g	674.33±4.2fg	15.25±0.08fg	26.07±0.16d	1712.7±11.54fg
	**AMF+Nitroxin**	1.26±0.004fg	724.51±3.3ef	15.92±0.05ef	26.56±0.76d	1827.4±8.98f

Within a column, means ± SE with similar letters are not significantly different at 5% probability based on LSD test.

### Grain number per panicle

The simple effects of irrigation and bio-fertilizer treatments on the grain number per panicle of sorghum showed a significant difference at the 1% level while the interaction effect of these treatments on this trait was significant at the 5% level (Table 7). The grain number per panicle of sorghum plants decreased under stress conditions compared to non-stress but increased with the application of bio-fertilizers compared to their non-application. In this regard, the highest number of grains per panicle (963.06 ± 4.6) belonged to the simultaneous application of AMF and Nitroxin bio-fertilizers in non-drought stress conditions and the lowest grain number per panicle (593.9 ± 2.3) was obtained by the treatment where AMF and Nitroxin were not used under severe drought stress conditions (Table 8). Therefore co-inoculation with both bio-fertilizers under severe stress conditions increased the grain number per panicle by 21.99% compared to the non-application of bacteria and fungi under the same conditions.

### Panicle length

The simple effects of irrigation and the bio-fertilizer application on the panicle length of sorghum showed a significant difference at the 1% level while the interaction effect of these treatments on this trait was significant at the 5% level (Table 7). The highest panicle length (20.22 ± 0.12 cm) was observed in the treatment where there was the interaction effect of the simultaneous application of AMF and Nitroxin bio-fertilizers and non-stress (irrigation after 60 mm evaporation) treatment. The lowest panicle length (13.32 ± 0.27 cm) belonged to the interaction effect between the non-application of bio-fertilizers and irrigation after 180 mm evaporation (severe stress) (Table 8). Therefore the panicle length of sorghum was markedly decreased under moderate (14.96%) and severe stress conditions (31.15%). The use of both Nitroxin and AMF under severe stress increased the size of this attribute by 20.23% compared to non-inoculation under severe stress conditions.

### 1000-grain weight

The simple effects of irrigation and the application of bio-fertilizer treatments and the interaction effect of these treatments on the 1000-grain weight of sorghum showed a significant difference at the 1% level (Table 7). In this study, the highest 1000-grain weight of sorghum (39.11 ± 0.10 g) was obtained from the interaction effect of the simultaneous inoculation of AMF and Nitroxin and irrigation after the 60 mm evaporation treatment. There was no significant difference between the treatment of Nitroxin application under non-drought stress conditions (38.94 ± 0.38 g) and the treatment of inoculation with AMF in non-drought stress conditions (38.68 ± 0.11 g), in addition to the interaction effect of the non-application of bio-fertilizers and non-stress treatment (38.49 ± 1.10 g). However, the lowest value of this trait (22.09 ± 0.07 g) belonged to the non-application of AMF and Nitroxin bio-fertilizers in severe drought conditions (irrigation after 180 mm evaporation). Consequently, under severe drought stress conditions, the 1000-grain weight of sorghum was reduced by 42.60% compared to non-drought stress conditions but the co-inoculation of this plant with both Nitroxin and AMF under severe stress increased the amount of this trait by 20.23% compared to non-treated plants under severe stress (Table 8).

### Grain yield

In this study, the grain yield of sorghum was affected by irrigation stress and inoculation with bio-fertilizers at 1% level while the interaction effect of these treatments on this trait was significant at the 5% level (Table 7). According to the results of the means comparison (Table 8), grain yield was significantly decreased under drought stress conditions but significantly increased with the application of Nitroxin and AMF. In this regard, the highest degree of the grain yield trait (2,671.5 ± 12.98 kg ha^-1^) was obtained from the interaction effect due to the co-inoculation with AMF and Nitroxin bio-fertilizers and the treatment of irrigation after 60 mm evaporation (non-stress conditions). The lowest measurement of this trait (1,441.4 ± 6.06 kg ha^-1^) was obtained from the interaction effect of the non-application of these bio-fertilizers and irrigation after 180 mm evaporation (severe drought stress). Therefore the grain yield of sorghum under severe drought stress conditions was reduced by 43.77% compared to non-drought stress conditions. The application of both bacteria and fungi under severe stress elevated this trait by 27% compared to non-treated plants under severe stress.

## Discussion

The results from our experiment revealed a tripartite interaction between AMF, rhizobacteria and the host plant. We found that sorghum growth was clearly increased when the AMF was associated with rhizobacteria (Azospirillum and Azotobacter). This was in relation to the better growth of the plants under dual inoculation, suggesting the presence of a synergistic impact among the symbionts of the fungi and the strain of bacteria in the rhizosphere. AMF and rhizobacteria work together in the soil and plant tissues to increase plant growth by enhancing nutrition, facilitating the hyphae penetration of the plant roots, promoting rhizobacterial survival and protecting against biotic and abiotic stresses. Impressive molecular work has shown there to be a range of fundamental principles that underlie the interactions between plants and microbes such as:

- Microbial signals that the plant immune receptors understand and use to activate defensive or symbiotic reactions.
- Secretion systems of protein and/or microbial DNA that transport molecules to modulate the cell functions in the host plant cell [34].

Therefore another principal finding is the signaling among the plants and microorganisms via transport signaling compounds. To control these interactions, communication through signaling molecules such as flavonoids, strigolactones and sesquiterpenes is essential. It is known that strigolactones released in low concentrations by microorganisms of the rhizosphere promote the colonization of plants by AMF [35]. Increased plant growth under the conditions of synergistic interaction among PGPR and fungi compared to a single inoculation has been reported by other researchers [36]. The reduction in the content of chlorophyll (a, b, and total) in sorghum leaves under irrigation after 120 and 180 mm evaporation conditions (severe and moderate drought stress) compared to irrigation after 60 mm evaporation treatment (non-stress) is probably due to the oxidative damage to the chloroplast lipids (membrane lipids), pigments and proteins. It might also be related to an increase in chlorophyllase enzyme activity under drought stress conditions [37]. These results are in agreement with those found by Nxele et al. [8]. Moreover, in our study, increasing the chlorophyll content under drought stress through the inoculation with bio-fertilizers was probably due to the fact that bio-fertilizers play an important role in chlorophyll formation by increasing the pyridoxal enzyme activity. This enzyme plays an important role in the synthesis of α-aminolevulinic acid as a major compound in chlorophyll [38]. Consistent with these results, Rahimi et al. [23] also reported that the highest content of chlorophyll in cephalaria was obtained through the interaction of AMF and Azotobacter. In the present experiment, the reason for the reduction in the total soluble protein under drought stress conditions may be due to the negative effect of drought stress on the activity of the nitrate reductase enzyme (as an essential enzyme in protein synthesis) through the impact on the nitrate reductase gene transcription. It is also due to the reaction of ROS with proteins and the increasing activity of protein degrading enzyme under drought stress conditions which changes amino acids [39]. In our study, the sorghum leaf protein content was significantly increased under the application of AMF and Nitroxin bio-fertilizers, especially under drought stress conditions. Any increase in nitrate reductase enzyme activity, higher nitrogen fixation and growth hormone production through the application of these bio-fertilizers led to an increase in the leaf protein content of sorghum, especially in the drought stress treatments. These results have been confirmed by other researchers [40, 41].

In our study, the results show that the sorghum plants inoculated with bio-fertilizers had a higher RWC and lower WSD in all irrigation treatments in comparison with those that were not inoculated. It is logical that the relative water content of the sorghum is reduced when there is a higher amount of evaporation from the evaporation pan. The relative water content of a leaf indicates the amount of water in the plant and the ability of the plant to survive under conditions of drought stress [42]. This is directly related to the cell turgidity and the water potential of the plants. On the other hand, cell development and cell division are related to the cell turgid, and there is a close relationship between plant yield and leaf relative water content [43]. These results are in agreement with those found by Nxele et al. [8]. In our study, the improvement in the water relationship of mycorrhizal plants is probably a result of the higher water uptake due to changes in the root morphology stemming from the expansion of the surface absorbing area of the roots by mycorrhiza hyphae [44]. These results are in agreement with those found by Darbani et al. [10].

From the results, water use efficiency decreased with the increase in water supply and there was also a reduction in yield for the plants that were not inoculated with bio-fertilizers. Some studies have revealed an increase in WUE under drought stress conditions [45]. The higher water use efficiency in drought stress conditions is because drought-stressed sorghum plants wilt far more than plants under non-stress conditions. Wilting always happens in periods when the atmospheric saturation deficit is significant. Thus the plant only assimilates when the saturation deficit is minimal. Less water is lost for each stabilized carbon molecule as a result [46]. As the research results have shown, plants that had been inoculated with bio-fertilizers use less water per unit of dry matter than non-inoculated plants and there are also higher values of WUE in inoculated sorghum plants than non-inoculated plants. This may be because of the symbiotic relationship between the rhizobacteria and fungi that has increased the ability of the roots to absorb soil moisture and therefore kept opened the stomata in the leaves and increased the dry matter production. The enhancement of water conductivity may also be related to the increase in the surface area of the roots for the uptake of water and P and the higher osmoregulation provided by the AMF and rhizobacteria in soil [47].

Increased WUE values by inoculation with AMF and Azospirillum under drought stress conditions were reported for *Triticum aestivum* [48], *Leymus chinensis and Hemarthria altissima* [49]. In the present study, it was observed that the proline content was higher for the severe and moderate drought stress treatments than non-stress treatment. Proline is an important osmolality that is produced in many plant species in response to stress. The accumulation of proline in plant tissues through osmotic regulation provides the necessary energy for plant survival and growth. It can also control water entry and exit in the cytoplasm and vacuoles under drought stress conditions [50]. Furthermore, the increase in proline content at the time of Nitroxin and AMF application was probably due to the role of these bio-fertilizers in increasing the expression of the delta-1-pyrroline-5- carboxylate synthetase (as an effective enzyme in proline synthesis) gene [51]. This has been confirmed by the results of the previous research [11]. The results of Javan Gholiloo et al. [20] also show that the interaction effect of Mycorrhiza, Azotobacter and Azospirillum increases the proline and valerian tolerance to drought stress.

Membrane permeability is usually evaluated as membrane electrolyte leakage. This is a key indicator of the cell membrane health of plants under stress conditions [52]. Abiotic stress such as drought leads to the disruption of lipids and membrane proteins and eventually to changes in enzymatic activities [51]. Cell membrane stability is one of the most important parameters of the plant cell response and abiotic stress species tolerance in crop plants under drought stress conditions [53]. All types of reactive oxygen can react with DNA, proteins, and lipids [54]. In the absence of a protective mechanism, these free radicals can damage the cell wall, resulting in cell leakage and the impaired function of the plant [55]. An increase in cell membrane leakage and therefore a decrease in cell membrane stability under drought stress has been reported by some researchers [56]. In the present study, severe and moderate drought stress decreased membrane stability and increased the cell membrane leakage of sorghum plants. However, the application of bio-fertilizers under drought stress conditions significantly reduced the level of cell membrane leakage. The application of bio-fertilizers, probably through their impact on glutathione S-transferase-related proteins, plays a special role in protecting the cell membranes from damage and degradation by ROS [57]. Consistent with these results, the reduction of cell membrane leakage in *Elymus nutans* under the influence of AMF application has previously been reported by Chu et al. [58]. In our research, drought stress in sorghum plants obviously decreased the N content. Nitrogen is a vital mineral nutrient for the growth of crops and to ensure the high productivity of plants, it is of special importance to better realize the metabolism of N and the usage of plants in response to a water shortage [59]. The concentration of chlorophyll and the leaf color of the plants are both directly related to the amount of N. The yield and growth of plant are therefore reduced under drought stress conditions and an N shortage [60]. Research has shown that reductions in plant nitrogen content may be associated with decreases in the activity of nitrate reductase, nitrite reductase and glutamine synthetase under stress conditions [61]. Previous studies have also shown that a higher N metabolism increases crop drought tolerance [62]. In the present study, inoculation with AMF and Nitroxin (Azospirillum + Azotobacter) increased the N concentration in the plant tissues under both drought stress and non-stress conditions. Compared with a single inoculation treatment, dual inoculation with AMF and Nitroxin resulted in a higher N content. Several studies have shown that improving the uptake of nutrients using AMF and rhizobacteria is a basic mechanism for improving the adverse effects of drought stress in plant development [20, 63].

Generally in the host plant, AMF increases the N level in two ways: direct (soluble nitrogen absorption and transfer) and indirect (through the insoluble organic compounds releasing and converting the insoluble nitrogen in the soil into a soluble nitrogen phase and subsequently transferring it), in addition to the influence of the fungi on the nitrogen process [64]. These results are consistent with Shi et al.’s [13] results. In addition to molecular nitrogen stabilization, rhizobacteria present in the Nitroxin bio-fertilizer induce the production of the IAA hormone. This increases the length of the root plant and therefore improves the uptake of N from the soil [65]. The synergistic effects of fungi and N-fixing bacteria in terms of increasing the NR, NiR and GS enzymes activities in sorghum plants inoculated with both fungal and Nitroxin bio-fertilizers can explain the increase in the N content of the plant [66, 67].

The results of our research show that AMF spore density decreased with higher levels of drought stress. Similar studies showed that drought stress decreased AMF spore population. In this context, water deficit stress probably inhibited the germination of spores and the growth of mycelia in the rhizosphere of the host plant while limiting the transmission of infection from the spores [68]. The reduction in root secretions which occurred due to lower plant photosynthesis was another reason for the decrease in AMF spore number by drought stress [69]. The results of our experiment revealed that the level of sorghum root colonization in inoculated sorghum plants with bio-fertilizers was significantly higher than those of non-inoculated plants. Furthermore, the Nitroxin treatment showed no AMF spore number because both of the microorganisms (Azotobacter and Azospirillum) belong to the non-infectious group and are mycorrhizal-activating microbes. This is consistent with the experimental evidence reported by Russo et al. [70] who found that Azospirillum genus bacteria were attached to the surface of structures such as mycelia, fungal spores and roots. This indicates that bacteria can use fungal structures as bridges to reach deep into the radical epidermis. In addition, it is proposed that Azospirillum and Azotobacter increase AMF-plant symbiosis because of the phytohormone production that stimulates root branching. Carina et al. [71] suggested that the physiologically and enzymatically active mycelium results in newly-formed lateral roots since AMF-bacteria develop a functional interaction with the mycorrhizosphere. Finally, the increase in both microorganism populations is evidence of a synergistic relationship. Improvements in the AMF spore count under the combined inoculation of AMF+ Azospirillum was reported by Patil and Santhosh [22]. Therefore further spore density in the soil due to dual inoculation treatment can be attributed to the positive interaction of AMF and dinitrogen-fixing bacteria. This can be related to the production of plant growth substances and the enhancement of the auxin, cytokinin and gibberellin excretions by the rhizobacteria [72].

The results of our experiment showed that with the increase in irrigation intervals, the number of panicles per sorghum plant significantly decreased, probably due to the decrease in the production and transportation of photosynthetic materials under drought stress conditions. The inoculation of sorghum seeds with bio-fertilizers increased the length and density of the hairy roots, thereby increasing the water and nutrient uptake and production of growth hormones such as auxin. In addition, by improving the net photosynthesis, the number of panicles of the sorghum plant increased [73]. Saed Moucheshi et al. [74] also reported an increase in the number of wheat spikelets under drought stress and mycorrhizal symbiosis conditions. In the present study, the decrease in the number of grains under moderate and severe drought stress conditions was probably due to the dehydration of the pollen grains and lack of proper pollination [75]. Consistent with these results, a decrease in the number of sorghum grains under drought stress was reported by Jabereldar et al. [76] and Oliveira Neto et al. [77]. In our study, the simultaneous use of AMF and Nitroxin in drought stress conditions increased the plant’s access to nutrients and prevented contamination with plant pathogens in addition to improving its growth characteristics, consequently increasing both the yield and yield components [78]. The positive effect of these bio-fertilizers on the number of grains per panicle of plants has been confirmed by Farnia and Hadadi [11]. Panicle length has a major role in the number of grains per panicle and the final yield of the plants. We observed that with the increasing evaporation level from 60 to 180 mm from the pan surface, the panicle length of sorghum significantly decreased. Drought stress reduces cell elongation more than cell division and it also affects various physiological and biochemical processes such as photosynthesis, respiration, the transport and uptake of ions, carbohydrates, the metabolism of substances and hormones and the growth of plant organs including panicle length. Consistent with these results, a reduction in the panicle length of sorghum under drought stress was also observed in the results of Jabereldar et al. [76]. In our study, the positive effect of AMF and Nitroxin bio-fertilizers in terms of preventing the grain size reduction of sorghum, especially under drought stress, was observed. To explain, the mycelium propagation of AMF and the formation of an additional uptake system complementary to the plant root system [79], as well as indoleacetic acid along with the cytokinin produced by the bio-fertilizers, might increase the length of the panicles by increasing the leaf weight [80]. Consistent with these results, in the study by Patil and Santhosh [22], the highest panicle length and number of filled and unfilled grains per panicle of rice plants were obtained following the interaction of AMF and Azospirillum compared to the control and individual inoculations.

In the present study, the reduction of the 1000-grain weight of sorghum in 120 and 180 mm evaporation conditions (moderate and severe drought stress) compared to 60 mm evaporation (non-stress) was probably due to water stress affecting the stomatal opening process, resulting in the reduction in the activity of Calvin cycle enzymes and the amount of assimilate production [81]. The shortening of the grain filling period under drought stress conditions is another reason for the loss of the 1000-grain weight in stress treatments [82]. According to these results, 1000-grain weight losses of sorghum and sunflower under drought stress conditions were reported by Oliveira Neto et al. [77] and Dehkhoda et al. [83]. It seems that inoculation with bio-fertilizers, with an increased speed and duration of photosynthesis, heightened dry matter accumulation and elevated material transfer efficiency to grains, leads to an increase of 1000 grain weight and the grain yield of sorghum [84]. Consistent with these results, Farnia and Hadadi [11] also reported an increase in the 1000-grain weight of maize after AMF inoculation under non-stress and drought stress conditions.

In the Javan Gholiloo et al.’s [20] experiment on the synergistic relationships between mycorrhiza, Azotobacter and Azospirillum, there were positive effects on the yield and tolerance of the valerian to drought stress. In our experiment, the reduction in the grain yield of sorghum under irrigation after 120 and 180 mm evaporation (moderate and severe drought stress) conditions compared to irrigation after 60 mm evaporation (non-stress) treatment was probably due to the lower chlorophyll content. This causes a reduction in the allocation of photosynthetic materials to the plant organs and as a result, the abortion and drying of flowers increases [85]. These results are also in agreement with those found by Jabereldar et al. [76]. The results of our experiment show that the application of both bio-fertilizers (AMF and Nitroxin) under drought stress and non-stress conditions significantly increased the grain yield of sorghum. This increase was more pronounced under drought stress conditions. It seems that the increased grain yield of sorghum in response to inoculation with these bio-fertilizers is related to the availability of nutrients for the growth of the plant which, in turn, increased the production of photoassimilates for grain filling. Microorganisms in bio-fertilizers can improve the growth of a plant through nitrogen fixation or the production of growth hormones [86]. Therefore a synergistic relationship promotes the growth and yield of plants because microorganisms allow the plants achieve greater absorption of phosphorus, nitrogen and other elements than those treated with a single inoculation and the control [18]. The enhancement of plant biomass by the co-inoculation of AMF and bacterial strain in comparison with AMF inoculation alone has already been reported [11, 16, 22].

## Conclusions

Moderate and severe drought stress treatments caused by irrigation after the evaporation of 120 and 180 mm water from an evaporation pan reduced both the yield and yield components of grain sorghum. However, the co-inoculation with Nitroxin and AMF proved to be useful in terms of mitigating the negative effects of drought stress by improving physiological traits such as chlorophyll, protein, proline, N content in plant and the RWC content as well as by decreasing the electrolyte leakage and WSD which led to a higher yield and improved yield components of the plant. Under drought stress conditions, there was a 27% increase in grain yield under the synergistic effects of bacteria and fungi compared to the non-application of these microorganisms As a result, the inoculation of plants with Azospirillum and Azotobacter bacteria and AMF is an effective method to alleviate drought stress in the plant’s responses, thus maintaining the grain yield and yield components during water stress for sorghum. In the present study, we observed a significant growth benefit from the synergistic association of sorghum with AMF and rhizobacteria under drought stress. Therefore the application of Nitroxin and AMF for sorghum growth is recommended in the Saravan region and other areas with similar climates.
